# The effect of an enriched environment on activity levels in people with stroke in an acute stroke unit: protocol for a before-after pilot study

**DOI:** 10.1186/s40814-016-0081-z

**Published:** 2016-08-02

**Authors:** Ingrid C. M. Rosbergen, Rohan S. Grimley, Kathryn S. Hayward, Katrina C. Walker, Donna Rowley, Alana M. Campbell, Suzanne McGufficke, Samantha T. Robertson, Janelle Trinder, Heidi Janssen, Sandra G. Brauer

**Affiliations:** 1 Division of Physiotherapy, School of Health and Rehabilitation Sciences, University of Queensland, Brisbane, Australia; 2 Allied Health Services, Nambour General Hospital, Sunshine Coast Hospital and Health Service, PO Box 547, Nambour, 4560 Australia; 3 Sunshine Coast Clinical School, The University of Queensland, Nambour, Australia; 4 Department of Physical Therapy, University of British Columbia, Vancouver, BC Canada; 5 Nursing and Midwifery, Sunshine Coast Hospital and Health Service, Nambour, Australia; 6 Hunter Medical Research Institute, Newcastle, Australia

**Keywords:** Stroke, Acute stroke unit, Enriched environment, Activity, Behavioral mapping, Complications

## Abstract

**Background:**

Clinical practice guidelines advocate engaging stroke survivors in as much activity as possible early after stroke. One approach found to increase activity levels during inpatient rehabilitation incorporated an enriched environment (EE), whereby physical, cognitive, and social activity was enhanced. The effect of an EE in an acute stroke unit (ASU) has yet not been explored.

**Methods/design:**

We will perform a prospective non-randomized before-after intervention study. The primary aim is to determine if an EE can increase physical, social, and cognitive activity levels of people with stroke in an ASU compared to usual care. Secondary aims are to determine if fewer secondary complications and improved functional outcomes occur within an EE. We will recruit 30 people with stroke to the usual care block and subsequently 30 to the EE block. Participants will be recruited within 24–72 h after onset of stroke, and each block is estimated to last for 12 weeks. In the usual care block current management and rehabilitation within an ASU will occur. In the EE block, the ASU environment will be adapted to promote greater physical, social, and cognitive activity. Three months after the EE block, another 30 participants will be recruited to determine sustainability of this intervention. The primary outcome is change in activity levels measured using behavioral mapping over 12 h (7.30 am to 7.30 pm) across two weekdays and one weekend day within the first 10 days of admission. Secondary outcomes include functional outcome measures, adverse and serious adverse events, stroke survivor, and clinical staff experience.

**Discussion:**

There is a need for effective interventions that starts directly in the ASU. The EE is an innovative intervention that could increase activity levels in stroke survivors across all domains and promote early recovery of stroke survivors in the acute setting.

**Trial registration:**

Australian New Zealand Clinical Trial Registry, ANZCTN12614000679684

## Background

Stroke survivors who receive care in an acute stroke unit (ASU) are more likely to be alive and independent compared with general ward care [1]. Characteristics of the ASU considered to contribute to these outcomes include early mobility and multidisciplinary coordinated rehabilitation [2] to prevent immobility-related complications [3] and commence functional recovery early after stroke. Strong evidence indicates that increased engagement in physical activities targeting mobility and arm-hand function early after stroke result in improved functional outcomes [4]. Yet, despite awareness of the positive effects of increased physical activity, available evidence indicates that stroke survivors spend the majority of the day physically inactive and alone early after stroke [5].

Social support has been recognized as an important determinant of health-related quality of life in stroke survivors [6]. The relationship between various types of social support such as emotional, instrumental, or informational support and quality of life is inconsistent [6]. Some studies have found that high levels of social support are associated with larger improvements in functional status [7, 8]. It is argued that social support can offer encouragement, assistance, and increase compliance with treatments [7, 9] and assist in dealing with the consequences of stroke [6]. However, not all aspects of social support may be beneficial; instrumental support may lead to poorer health if someone becomes dependent on the provided assistance [10]. Limited evidence is available regarding cognitive activity after stroke. Cognitive activity such as listening to music during early recovery has been shown to enhance focused attention [11], lessen depressed mood [11], and improve visual attention in those with unilateral neglect [12]. Despite these possible benefits, little is known regarding social and cognitive activity levels in stroke survivors early after stroke.

There is a need to identify interventions that can increase activity levels across physical, social, and cognitive domains and concurrently have a positive effect on outcomes early after stroke. One possible intervention is an enriched environment (EE). In animal research, an EE is defined as an organized stimulating environment to enhance sensory, motor, and cognitive activities [13]. A systematic review and meta-analysis in animal research of stroke has shown that an EE has a positive effect on physical recovery, learning, and exploratory behavior, which includes reduced decline in memory and levels of anxiety [14]. A pilot study of EE in people with stroke was recently undertaken in the sub-acute inpatient rehabilitation setting [15]. This study showed promising results, with increased activity levels demonstrated across these activity domains [15]. An EE was achieved within this study by creating communal areas with stimulating equipment and individual enrichment through provision of personal equipment such as music and hobby activities [15]. An EE can provide activities that are meaningful and tailored to each stroke survivor as a wide variety of activities can be included in an enriched clinical setting. In addition, novel advanced technology such as virtual reality, IPads, and active gaming technologies can be a valuable addition to provide stimulation to stroke survivors with a therapeutic effect [16–18]. Enrichment strategies with a more conventional character could involve music and art, as these activities have shown to reduce boredom and a positive effect on mood in stroke survivors [19, 20].

Taken together, it is plausible that the implementation of an EE immediately post-stroke within an ASU could positively influence activity levels across all domains and lead to fewer complications and improved functional outcomes. This pilot study aims to determine the effect of implementing an EE in an ASU on physical, social, and cognitive activity levels, adverse events, and functional outcomes. We hypothesize that stroke survivors who start their journey in an ASU with an embedded EE will be more active, experience fewer complications, and achieve greater functional outcomes compared to those who start their journey in an ASU without embedded EE.

## Methods/design

The study involves a prospective non-randomized before and after design. We will recruit 30 people with stroke to usual care (block 1) and subsequently 30 to EE intervention (block 2). We will evaluate activity levels across all domains, secondary complications, and functional outcomes within both blocks. Following block 1, we will embed an EE in the same ASU during a 6-week period before commencement of block 2. To determine if embedding an EE within an ASU persists, activity levels will be re-evaluated 3 months post-Block 2 (EE) with an additional 30 stroke participants.

### Participants and setting

All recruitment for this study will be conducted in the same ASU in a regional Australian acute hospital. The ASU is an endorsed unit with 470 stroke admissions per annum, an average acute length of stay of 4.1 days, and an in-hospital mortality rate of 17 %. Rehabilitation by a multidisciplinary team is commenced on the day of admission, with transfer to general inpatient rehabilitation units (public and private) if length of stay is predicted to be greater than 7–10 days. Several community-based rehabilitation services are available spanning home and center based and slower transition care. The ASU has 8 funded stroke beds and is embedded within a 16-bed ward (8 single rooms and 4 double rooms). The ward is supported by 2.0 fulltime equivalent (FTE) physiotherapists, 1.6 FTE occupational therapists, 1.0 FTE speech therapist, 1.0 FTE social worker, 0.5 FTE dietician, and 0.7 FTE therapy assistant.

All stroke survivors admitted to the hospital will be screened for eligibility with consecutive recruitment of eligible participants. Recruitment is estimated to occur over 12 weeks or until the target number is reached. Participants will be included if they are (1) admitted to the ASU within 24–72 h after onset of stroke (ischemic or hemorrhagic, first and/or recurrent stroke), (2) able to complete a transfer from bed to chair with assistance of two persons or less, (3) able to follow single stage commands, (4) requiring assistance for basic activities of daily living (ADLs), and (5) were able to walk independently premorbidly (functional ambulation category score ≥4) and have a modified Rankin Score (mRS) of ≤2 from self-report. Participants will be excluded if they have (1) a retrospective premorbid mRS of ≥3, (2) a concurrent diagnosis of rapidly deteriorating disease, or (3) have an extensive psychiatric history. To allow for observational data collection, stroke survivors will also be excluded if discharge from the ASU is likely to occur within 2 days of admission. Informed consent will be obtained from participants or their substitute decision maker. Participants will be informed that the project aims to compare an alternative model of rehabilitation with the traditional model of rehabilitation, but not informed regarding the specific intervention being investigated or group allocation.

### Baseline measures

Baseline measures will include demographics; previous mRS and living arrangements; stroke details such as date, estimated time of onset, lesion location and type, Oxford Stroke Classification, and National Institute of Health Stroke Scale (NIHSS). Stroke severity will be classified according to NIHSS [21] on admission (day 1 if thrombolyzed): mild (<8), moderate (8–16), and severe (>16).

### Intervention

In the 12-week usual care block, participants will receive usual stroke management within the ASU and rehabilitation will be delivered in one-on-one interventions by therapists to stroke participants. At this site, therapists have access to a common therapy room, discipline-specific allied health assistants for individual treatment sessions and equipment to increase practice. Staffing levels will be monitored across the study period to ensure that they remain consistent across blocks.

After the usual care block, there will be a 6-week period in which the environment of the ASU will be adapted. Several communal areas will be created on the ward where participants have access to stimulating equipment. Self-directed exercise programs, iPads loaded with apps, iPods loaded with music, books, board games, puzzles, magazines, newspapers, and music will be available during and outside of therapy hours. During these 6 weeks, staff focus towards enabling activity will be reinforced through interdisciplinary education sessions. At these sessions, the EE theoretical concept, EE intervention, and ‘enablers and barriers’ of implementation of the intervention will be interactively discussed [22]. We will educate staff to encourage participants to attend communal areas and to use stimulating equipment in communal areas and at the bedside. Allied health assistants will be trained from discipline-specific to “generic” allied health assistants for the EE intervention. By doing this, all assistants will be able to mobilize patients and assist all therapists. In addition to interdisciplinary education, we will appoint nurse champions to facilitate and encourage adherence to the intervention on a day-to-day basis.

In the 12-week EE block, communal areas will be used to enhance individual and group activities. A daily group session will focus on different aspects of stroke recovery such as education, emotional support, communication, and physical activities. On three weekdays, there will be an interactive breakfast, and every weekday, an interactive lunchtime will be organized. These interactive mealtimes are aimed to increase the frequency of mobilization, encourage sitting upright for mealtimes, and stimulate social interaction. Staff present during mealtimes will facilitate independence in consuming meals and encourage nutritional intake in participants. Participants will be encouraged to voluntarily attend mealtimes without coercion for any activity. As total staffing numbers will not change with the EE, any staff time in group/meal sessions will be diverted from previous 1:1 therapy time. Allied health assistants will play the main role in managing mealtimes.

Participants of the EE block will receive a brochure that outlines the importance of frequent activity early after stroke; advice about how families can be involved and the day structure of the ASU will be explained. To encourage family involvement, stimulating equipment and individualized activity cards will be placed at the participant’s bedside tailored to the stroke survivor’s goals. Family and staff will be advised to bring personal items and hobby activities for the participant and to encourage the participant to engage in these activities outside therapy hours and on weekends.

### Fidelity of the intervention

During the EE intervention, we will monitor the occurrence of mealtimes and group sessions, availability of resources, and provision of information brochures. Our main measure for a successful implementation is to determine if participants in the EE intervention demonstrate significant higher activity levels as compared to usual care and that the EE is a safe intervention in the ASU.

### Primary outcomes

Activity levels will be determined for 'any activity', physical, social, and cognitive activity and time spent alone. Any activity is defined as the stroke survivor performing at least one physical, social, or cognitive activity or a combination of activities in these domains [23]. Total activity will be expressed as a percentage of the total number of observations performed, as will activity within physical, social, and cognitive activity domains. The first 10 days after admission will be considered as the primary exposure profile.

The behavioral mapping protocol by Janssen et al. [23] has been adapted for this study and will be used for measuring activity. Protocol adaptation included incorporating typical activities and equipment utilized in the acute setting.

Participants will be observed for 1-min at 10-min intervals from 7.30 am till 7.30 pm on two weekdays and one weekend day for a maximum of three mapping days or until discharge from the ASU. For each observation, the main activity performed during 1-min will be recorded for each category. In addition, we will report if the participant performed the observed activity independently, supervised, or with assistance. The participant’s location, body position, and people present will also be documented. Participants can be engaged in 'no activity' and can perform activities across more than one domain concurrently.

During each observation, the observer will collect data for as many categories as able. When the observer is unable to view the participant due to activity precluding direct observation (e.g., participant is in the bathroom), an attempt will be made to retrospectively estimate activity from nearby staff or the participant. Intervals where a participant is unable to be observed (e.g., off the ward for investigations) or activity unable to be estimated will be classified as 'unobserved'. Unintentional non-observations will be classified as 'missing' data. Unobserved and missing data will not contribute to the total number of observations for a participant. The reason for and proportion of 'unobserved and missing observations' will be reported by group.

Staff performing behavioral mapping will have specific training, followed by assessment compromising observation of four patients for 1 h, providing 24 observations. Competency to record study data is defined as attaining ≥90 % agreement with concurrent observations by the investigator. Behavioral mapping staff members will not receive any study details.

### Secondary outcomes

Secondary measures to be collected include functional outcomes, adverse and serious adverse events, and mood. Functional outcomes will be assessed with the mRS, modified Barthel index (MBI), 10-m walk test, and Mobility Scale for Acute Stroke patients (MSAS). Mood will be assessed with the Hospital Anxiety and Depression Scale (HADS), and nutritional status will be determined through weight and the Subjective Global Assessment (SGA) to assess for malnutrition. Serious adverse events (SAE) are defined as an adverse event that led to death and/or led to serious deterioration in health of a patient, whereas adverse events (AE) are defined as any untoward or unfavorable medical occurrence in a patient. Complications recorded include falls, pneumonia, pressure areas, cardiac problems, seizures, reduced Glasgow Coma Scale, stroke, transient ischemic attack, urinary tract infection, depression, constipation, malnutrition, delirium, and “other” including shoulder pain, deep venous thrombosis, and urinary retention [24, 25].

### Data collection time points

An investigator will conduct the initial assessment on entry to the study. Blinded assessors will perform discharge assessments immediately prior to discharge from the ASU or when a decision for palliative intent is made. Blinded assessors will also undertake follow-up phone calls at 3 months post-stroke to determine mRS, living arrangement, Health State Score [26], and if any SAEs have occurred after discharge. Three months after the EE block, another period of behavioral mapping will occur to determine whether activity levels have been sustained in the ASU. We aim to recruit 30 participants using the same eligibility criteria and complete behavioral mapping on a single, randomly chosen day from 7.30 am till 7.30 pm.

### Participant and staff experience

**Fig. 1:**
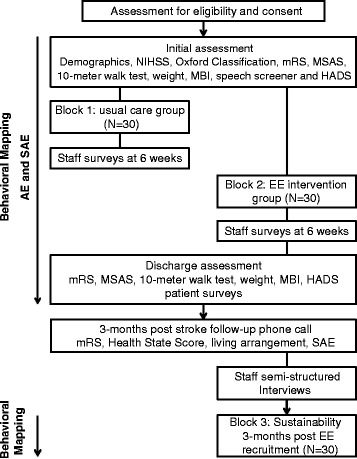
Flowchart of study. *NIHSS* National Institutes of Health Stroke Scale, *mRS* modified Rankin Scale, *MSAS* Mobility Scale for Acute Stroke, *MBI* Modified Barthel Index, *HADS* Hospital Anxiety and Depression Scale, *EE* enriched environment, *AE* adverse events, *SAE* serious adverse events

During both blocks, patient/carer and staff experiences will be explored through surveys. A short patient and carer survey will explore the patient/carer’s experience and whether they felt sufficient stimulation was offered to assist early rehabilitation and recovery during the acute stay. Staff surveys will be handed out to 25 nursing and therapy staff members of the unit. These surveys will explore their perceptions of innovation, patient care, team relationships, work satisfaction, and workload during each block. In addition, after block 2, up to ten semi-structured interviews with nursing and therapy staff will be conducted until saturation of data is reached. These interviews will focus on staff experience during the EE intervention period only. Questions will aim to explore perceived barriers in implementing an EE and what staff will need to be able to sustain the EE in the future. Thematic content analysis will be performed on all qualitative data. Figure 1 shows a flow chart of the pilot study.

### Sample size

We performed a sample size calculation to ensure that this pilot study has sufficient power to determine significant positive effect on our primary outcome measure—total activity levels in stroke survivors. Estimates were based on data from a recent study of the effect of an EE on activity levels conducted during inpatient stroke rehabilitation [15]. This study reported a mean increase in the proportion of observations with 'any activity' in the EE group of 13 % (SD 14) and the control (no EE) group of 2 % (SD 16.5), equivalent to an effect size of 0.719. We performed a one-sided, between-groups test as this past research has shown an enriched environment to increase activity levels in stroke survivors [15]. Based on a rounded effect size of 0.7, we calculated that we would need to recruit 26 participants per group to detect one standard deviation difference between EE and non-EE groups for any activity with an alpha level of 0.05 and a power of 0.8. Allowing for a conservative dropout rate of 12 %, we will aim to recruit 30 participants per group.

### Statistical analysis

Baseline characteristics of participants will be described using means and standard deviations for continuous variables and counts and percentages for categorical variables. To address the primary outcome (change in activity levels for 'any activity'), we will compare the proportion of activity between usual care and EE intervention group. We will assume that at each observation, the participant remains engaged in that activity for the entire 10-min interval; this will allow us to calculate the proportion of time each group is engaged in any activity. Subsequently, we will explore for differences in each activity domain and subcategories within domains. Consistent with previous research in the field [15], unobserved and missing data will be excluded from the analysis for the primary outcome of change in activity level. Total number of observations per participant will be summed and used to calculate the proportion of observations each participant is observed to be engaged in any activity (and physical, social, and cognitive activity and other fields included in the data collection sheet). The difference in activity levels between groups will be determined using one-way ANCOVA with group as independent variable, activity levels as the dependent variable, and adjusting for covariates age (years), stroke severity (NIHSS), and premorbid function (mRS). To determine differences in secondary outcome measures between groups, one-way repeated-measures ANCOVA will be performed for variables separately, adjusting for age (years), stroke severity (NIHSS), and premorbid function (mRS). We will adjust for the above covariates as these covariates impact on activity levels, functional outcomes, and mood in stroke survivors [27–29].

## Discussion

Research has consistently shown that ASU care provides very limited opportunities for people with a stroke to be involved in physical activities and that patients are often alone. Increased activity levels after stroke are associated with better functional outcomes and reduced complication rates. In addition, with the aging population and the rising incidence in stroke, there is a strong need for the development of resource-efficient interventions that can improve patient and service outcomes without increasing staffing cost. The EE is an innovative interdisciplinary model of care that could build the capacity of acute stroke teams to deliver efficient and effective care for people with stroke.
